# Enhanced Immunomodulatory Effects of Thymosin-Alpha-1 in Combination with Polyanionic Carbosilane Dendrimers against HCMV Infection

**DOI:** 10.3390/ijms25041952

**Published:** 2024-02-06

**Authors:** María de la Sierra Espinar-Buitrago, Esmeralda Magro-López, Elena Vázquez-Alejo, María Ángeles Muñoz-Fernández

**Affiliations:** 1 Section of Immunology, Immuno-Molecular Biology Laboratory (LIBM), University General Hospital Gregorio Marañon (HGUGM), 28007 Madrid, Spain; marisierri90@gmail.com (M.d.l.S.E.-B.); esmeraldamagrolopez@hotmail.com (E.M.-L.); elena2393.ev@gmail.com (E.V.-A.); 2 Gregorio Marañon Health Research Institute (IiSGM), 28009 Madrid, Spain; 3 Center for Biomedical Research in Bioengineering, Biomaterials and Nanotechnology Network (CIBER-BBN), 28029 Madrid, Spain; 4 HIV-HGM Biobank, University General Hospital Gregorio Marañon (HGUGM), 28007 Madrid, Spain

**Keywords:** polyanionic carbosilane dendrimers, G2-S16, G2-S24P, Thymosin-alpha-1, immunomodulation, human cytomegalovirus

## Abstract

Resistance and toxicity associated with current treatments for human cytomegalovirus (HCMV) infection highlight the need for alternatives and immunotherapy has emerged as a promising strategy. This study examined the in vitro immunological effects of co-administration of Thymosin-alpha-1 (Tα1) and polyanionic carbosilane dendrimers (PCDs) on peripheral blood mononuclear cells (PBMCs) during HCMV infection. The biocompatibility of PCDs was assessed via MTT and LDH assays. PBMCs were pre-treated with the co-administered compounds and then exposed to HCMV for 48 h. Morphological alterations in PBMCs were observed using optical microscopy and total dendritic cells (tDCs), myeloid dendritic cells (mDCs), and plasmacytoid dendritic cells (pDCs), along with CD4+/CD8+ T cells and regulatory T cells (Treg), and were characterized using multiparametric flow cytometry. The findings revealed that Tα1 + PCDs treatments increased DC activation and maturation. Furthermore, increased co-receptor expression, intracellular IFNγ production in T cells and elevated Treg functionality and reduced senescence were evident with Tα1 + G2-S24P treatment. Conversely, reduced co-receptor expression, intracellular cytokine production in T cells, lower functionality and higher senescence in Treg were observed with Tα1 + G2S16 treatment. In summary, Tα1 + PCDs treatments demonstrate synergistic effects during early HCMV infection, suggesting their use as an alternative therapeutic for preventing virus infection.

## 1. Introduction

Human cytomegalovirus (HCMV) infection is highly prevalent worldwide [1], being a major cause of morbidity and mortality among immunocompromised patients, such as AIDS patients or transplant recipients [2]. Currently, antiviral drugs used in the treatment of patients with HCMV infection have low oral bioavailability and dose-related toxicities, such as bone marrow suppression, hepatotoxicity and nephrotoxicity [3]. As a result, there is a great need for the development of alternative treatment options against HCMV [4,5]. In this regard, immunotherapy has emerged as a promising strategy to overcome the side effects of antiviral treatment and for the development of prophylaxis measures [6,7,8]. Particularly, Thymosin-alpha-1 (Tα1) has been shown to play a key role in the control of immunity, tolerance and inflammation, which explains its wide range of clinical applications in several pathologies, including infectious diseases [9]. Recently, we have studied the immunomodulatory effect of Tα1 in a SARS-CoV-2 scenario [10] and during HCMV infection, demonstrating that Tα1 treatment might support its use as an adjuvant for therapeutic treatment with cell therapies or with routine HCMV drugs in immunocompromised patients [11].

In addition, dendrimer-based molecules have been recognized as an effective immunotherapy for viral infections, cancer, and autoimmune diseases due to their ability to efficiently capture and load antigens, their biocompatibility, and their versatility in various therapeutic applications [12]. Specifically, polyanionic carbosilane dendrimers (PCDs) have shown their ability to prevent the transmission of several sexually infectious diseases, such as Human Immunodeficiency Virus (HIV-1), Human Herpes Virus type 1 or 2 (HSV-1/HSV-2) or Hepatitis C Virus (HCV) [13,14,15], and even HCMV, as we have previously demonstrated [16]. Additionally, some PCDs have been shown to exert action on several cells of the immune system, such as anti-inflammatory treatments [17] or by increasing regulatory T cells’ (Treg) capacity during HIV-1 infection [18].

Thus, the aim of this study was to evaluate the immunological effects of combined treatment with Tα1 and two PCDs (G2-S16 or G2-S24P) during early HCMV infection in vitro in order to determine if there could be a synergistic effect between both treatments, suggesting their use as a therapeutic alternative for the prophylaxis of HCMV infection.

## 2. Results

### 2.1. The G2-S16 and G2-S24P PCDs Exhibit Good Biocompatibility in PBMCs

**Figure 1:**
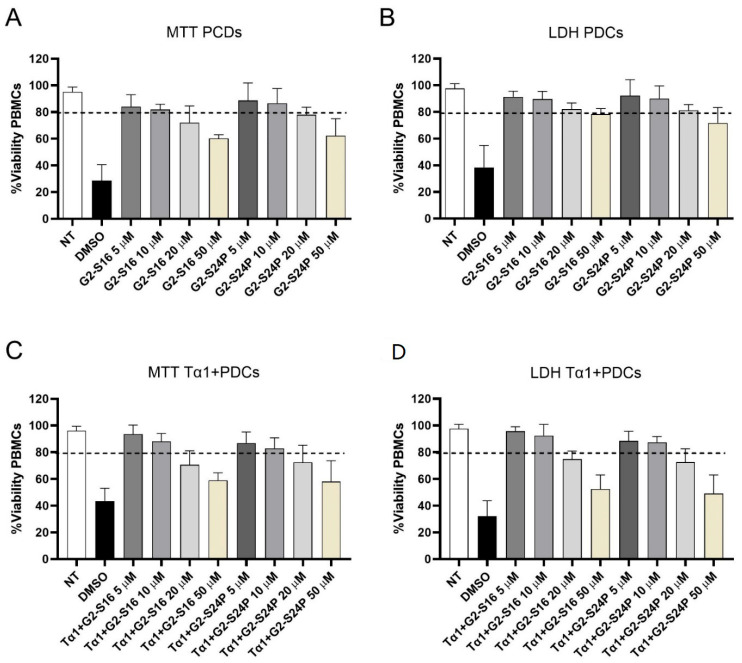
Biocompatibility studies of PCDs. PBMCs were treated with increasing concentrations (5–50 µM) of G2-S16 and G2-S24P PCDs for 48 h followed by MTT (**A**) and LDH (**B**) assays. Similarly, PBMCs were also treated with Tα1 (50 ng/mL) and increasing concentrations (5–50 µM) of G2-S16 and G2-S24P PCDs for 48 h followed by MTT (**C**) and LDH (**D**) assays. The mean values (mean ± SD) of at least three independent experiments are shown. Absorbance values were interpreted as a measurement of cell viability. Concentrations resulted in cell viability above 80%. Non-treated (NT) PBMCs were used as cell viability control. DMSO was used as a cell death control. Abbreviations: DMSO: Dimethyl sulfoxide; PBMCs: Peripheral Blood Mononuclear Cells; PCDs: Polyanionic Carbosilane Dendrimers, Tα1: Thymosin-alpha-1.

The biocompatibility analysis of G2-S16 and G2-S24P dendrimers in peripheral blood mononuclear cell (PBMC) cultures was conducted by MTT and LDH assays in treatments with increasing concentrations (5, 10, 20, and 50 µM) for 48 h (Figure 1). Concentrations where cell viability exceeded 80% were considered non-toxic in comparison to the non-treated condition [19]. The results demonstrated that G2-S16 and G2-S24P dendrimers exhibit good biocompatibility at concentrations of 5 and 10 μM, with cell viability values ranging between 80–85% for both MTT and LDH assays. The MTT and LDH results indicated that treatments with G2-S16 and G2-S24P PCDs for 48 h cause a dose-dependent reduction in mitochondrial activity but do not cause any damage in PBMC membranes categorical of cell death. Similar results were observed when MTT and LDH biocompatibility analyses were performed on Tα1 + PCDs co-treatments.

### 2.2. Tα1 + PCDs Treatments Mitigate Overactivation during HCMV Infection

**Figure 2:**
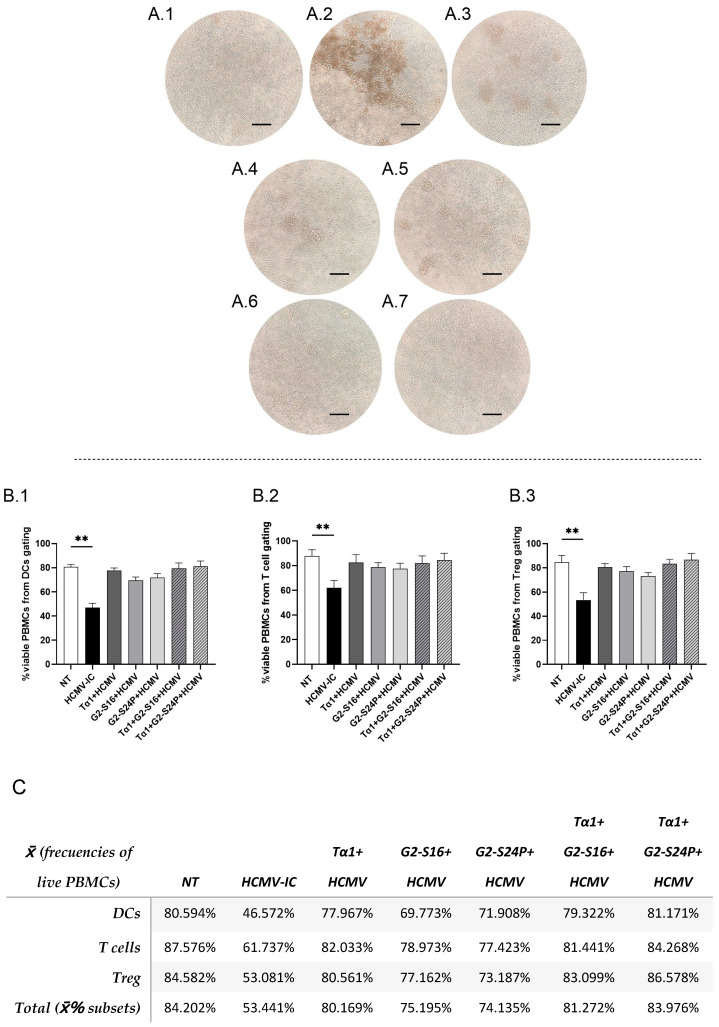
Representative microscopy images and viability of PBMCs in HCMV infection. PBMCs (300,000 cells) were pre-treated with Tα1, PCDs and their combinations, infected with HCMV for 2 h later and then cultured for 48 h in culture medium at 37 °C/5% CO_2_. Then, microscopy images were taken on a DMIL Leica microscope at 4X augmentation. NT: Non-treated control (**A.1**); HCMV-IC: HCMV Infection control (**A.2**); Tα1 + HCMV: Tα1 treated and HCMV infected condition (**A.3**); G2-S24P + HCMV: G2-S24P treated and HCMV infected condition (**A.4**); Tα1 + G2-S24P + HCMV: Tα1 + G2-S24P treated and HCMV infected condition (**A.5**); G2-S16 + HCMV: G2-S16 treated and HCMV infected condition (**A.6**); Tα1 + G2-S16 + HCMV: Tα1 + G2-S16 treated and HCMV infected condition (**A.7**). Bar graphs represent percentage of live PBMCs from total of events analyzed from DCs (**B.1**), T cells (**B.2**) and Treg (**B.3**) flow cytometry panels in the different study conditions. Table (**C**) resumes data shown in bar graphs, and the total percentage of viable PBMCs calculated as the average of the viability percentages of each cell population. The mean values (mean ± SD) of at least three independent experiments are shown (** *p* ≤ 0.01). Abbreviations: DCs: Dendritic cells; PBMCs: Peripheral Blood Mononuclear Cells; PCDs: polyanionic carbosilane dendrimers; HCMV: Human Cytomegalovirus, Tα1: Thymosin-alpha-1; Treg: regulatory T cells.

To explore the effects of the different treatments on cellular morphology in the presence of the virus, a PBMC culture was pre-treated with Tα1 and PCDs for 2 h and subsequently infected with HCMV for 48 h. The results revealed the presence of cellular aggregates in the infected control (HCMV-IC) (Figure 2(A.2)). Conversely, Tα1 treatment exhibited a reduction in both the size and number of aggregates, as did G2-S24P and Tα1 + G2-S24P treatments compared to HCMV-IC (Figure 2(A.3–A.5)). Similarly, G2-S16 and Tα1 + G2-S16 showed absence of morphological changes in PBMCs (Figure 2(A.6,A.7)). In addition, to determine whether the presence of aggregates was related to cell viability, a flow cytometry analysis was performed. PBMC viability was calculated as the number of viable PBMCs relative to the total of events recorded by the cytometer for each cellular population in the different conditions of the study.

These results showed a significant decrease in the percentage of PBMC viability in HCMV-IC condition compared to non-treated condition (NT) in dendritic cells (DCs) (Figure 2(B.1)), T cells (Figure 2(B.2)) or Treg (Figure 2(B.3)) flow cytometry panels. However, the percentage of viable PBMCs in all other conditions is similar to that observed in the NT (Figure 2C).

In addition, to determine whether the reduction of aggregates was associated with a decrease in virus presence, early viral protein (IE) was measured immediately by confocal microscopy analysis. The results indicated a reduced fluorescence intensity of IE in Tα1 + PCDs, as well as in Tα1, G2-S16, and G2-S24P alone, compared to HCMV-IC. Furthermore, a reduction in the fluorescence intensity of IE was observed between Tα1+ PCDs treatments and each PCD alone (Supplementary Figure S2).

### 2.3. Treatment of Tα1 with PCDs Enhances the Expression of Activation and Maturation Markers in DCs during HCMV Infection

To assess the immunological effects of treatment with Tα1 in combination with PCDs during HCMV infection, the expression of CD40, CD80, CD58, TIM-3 and HLA-DR mean fluorescence intensity (MFI) in DCs derived from PBMCs was analyzed using multiparametric flow cytometry. 

**Figure 3:**
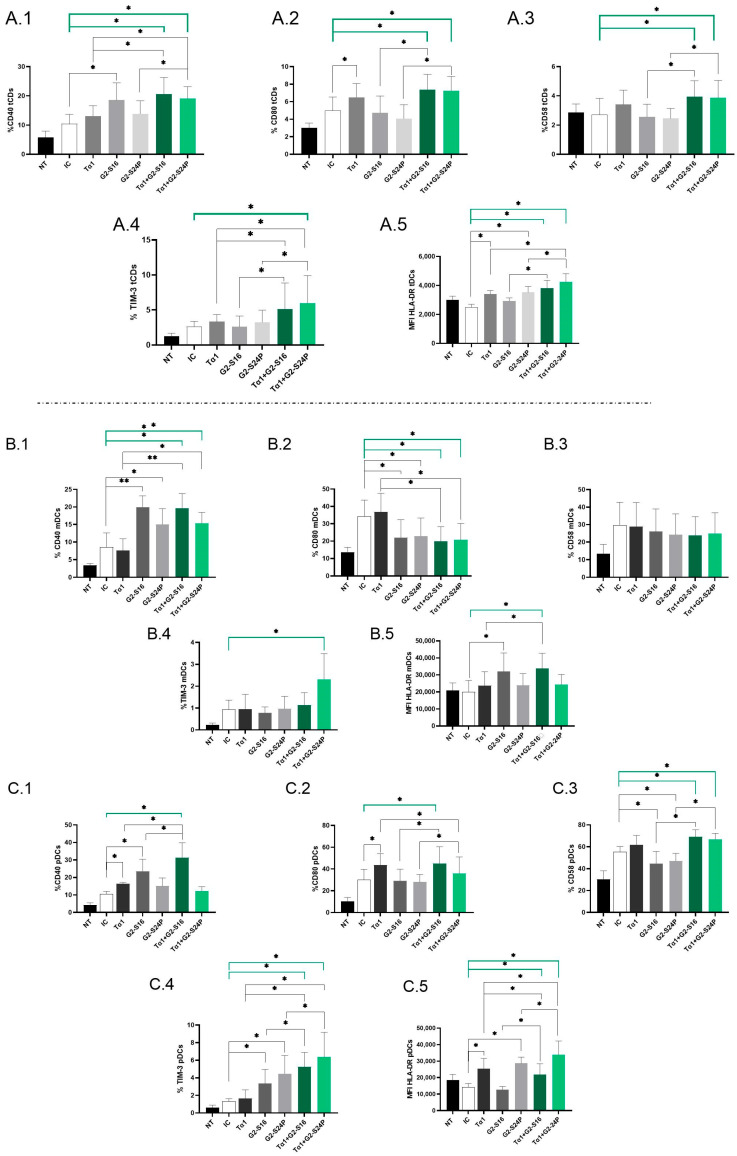
Immunophenotyping of DCs in Tα1 and PCDs co-treatment. PBMCs (300,000 cells) were pre-treated with Tα1, PCDs and their combination, infected with HCMV 2 h later and then cultured for 48 h in culture medium at 37 °C/5% CO_2_. The expression of activation and maturation markers were analyzed by multiparametric flow cytometry. Bar graphs represent the expression of each biomarker and HLA-DR MFI in tDCs: CD40 (**A.1**), CD80 (**A.2**), CD58 (**A.3**) and TIM-3 (**A.4**) and HLA-DR MFI (**A.5**); in mDCs: CD40 (**B.1**), CD80 (**B.2**), CD58 (**B.3**), TIM-3 (**B.4**) and HLA-DR MFI (**B.5**) and in pDCs: CD40 (**C.1**), CD80 (**C.2**), CD58 (**C.3**), TIM-3 (**C.4**) and HLA-DR MFI (**C.5**). NT: Non-treated control; HCMV-IC: HCMV Infection control; Tα1 + HCMV: Tα1 treated and HCMV infected condition; G2-S16 + HCMV: G2-S16 treated and HCMV infected condition; G2-S24P + HCMV: G2-S24P treated and HCMV infected condition; Tα1 + G2-S16 + HCMV: Tα1 + G2-S16 treated and HCMV infected condition; Tα1 + G2-S24P + HCMV: Tα1 + G2-S24P treated and HCMV infected condition. The mean values (mean ± SD) of at least three independent experiments are shown (* *p* ≤ 0.05; ** *p* ≤ 0.01. Abbreviations: PBMCs: Peripheral Blood Mononuclear Cells; PCDs: Polyanionic Carbosilane Dendrimers; HCMV: Human Cytomegalovirus; MFI: mean fluorescence intensity; Tα1: Thymosin-alpha-1; tDCs: total dendritic cells; mDCs: myeloid dendritic cells; pDCs: plasmacytoid dendritic cells.

Analysis of total dendritic cells (tDCs) revealed a significant increase in CD40 expression in both Tα1 + PCDs treatments compared to HCMV-IC. Notably, Tα1 + PCDs treatments showed elevated CD40 expression with respect to Tα1 alone, as well as an increased expression between Tα1 + G2-S24P treatment and G2-S24P alone. Moreover, higher CD40 expression was observed in G2-S16 treatment compared to HCMV-IC (Figure 3(A.1)). CD80 expression exhibited a similar trend, with both Tα1 + PCDs treatments showing increased levels compared to HCMV-IC and compared to each PCD treatment alone. Moreover, Tα1 alone demonstrated elevated CD80 expression compared to HCMV-IC (Figure 3(A.2)). Similar results were observed in CD58 expression (Figure 3(A.3)). Furthermore, TIM-3 expression was notably increased in the Tα1 + G2-S24P treatment compared to HCMV-IC and increased levels were observed between Tα1 + PCDs treatments and each individual Tα1, G2-S16 or G2-S24P treatment (Figure 3(A.4)). Additionally, increased HLA-DR MFI level was noted in both Tα1 + PCDs treatments, in G2-S24P alone and in Tα1 treatment compared to HCMV-IC. Similarly, an increase in HLA-DR MFI was observed between Tα1 + PCDs treatments and each PCD treatment alone, and between Tα1 + G2-S24P treatment and Tα1 alone (Figure 3(A.5)). No further changes were observed in tDCs for any studied marker and for HLA-DR MFI. 

Subpopulation analysis revealed a significant increase in CD40 and a decrease in CD80 expression in both Tα1 + PCDs treatments compared to HCMV-IC. Similar trends were observed in Tα1 + G2-S16 and Tα1 + G2-S24P treatments compared to Tα1 alone and between G2-S16 and G2-S24P compared to HCMV-IC in myeloid dendritic cells (mDCs) (Figure 3(B.1,B.2)). Nevertheless, no further changes were noted for CD58 or TIM-3 in mDCs, except for a significant increase in TIM-3 expression with Tα1 + G2-S24P treatment compared to HCMV-IC (Figure 3(B.3,B.4)). Furthermore, an increased HLA-DR MFI was observed in both Tα1 + G2-S16 and G2-S16 treatments compared to HCMV-IC. Moreover, higher HLA-DR MFI level was observed in Tα1 + G2-S16 compared to Tα1 alone (Figure 3(B.5)).

On the contrary, in plasmacytoid dendritic cells (pDCs), a significant increase in CD40 expression was observed in Tα1 + G2-S16 treatment compared to HCMV-IC and to Tα1 and G2-S16 alone, while no changes were observed with Tα1 + G2-S24P. Additionally, higher CD40 expression was noted in Tα1 and G2-S16 treatments compared to HCMV-IC (Figure 3(C.1)). For CD80 expression, an increase in Tα1 + G2-S16 treatment compared to HCMV-IC was observed. Similarly, Tα1 + PCDs treatments showed higher CD80 expression than each individual PCD treatment but a decrease between Tα1 + G2-S24P treatment and Tα1 alone was observed. Moreover, Tα1 treatment increased CD80 expression compared to HCMV-IC (Figure 3(C.2)). Furthermore, increased CD58 and TIM-3 expression in both Tα1 + PCDs treatments compared to HCMV-IC and to each PCD treatment alone were observed. However, while there was a decreased CD58 expression in PCDs treatments compared to HCMV-IC, increased TIM-3 levels were observed. Tα1 + PCDs treatments showed higher TIM-3 expression levels than Tα1 alone (Figure 3(C.3,C.4)). Higher HLA DR-MFI level with both Tα1 + PCDs treatments, G2-S24P alone and Tα1 were observed compared to HCMV-IC. Additionally, an increased HLA DR-MFI level between both Tα1 + PCDs treatments and each PCD alone were observed. Finally, an increased HLA-MFI level in Tα1 + G2-S24P and a decreased HLA-MFI level in Tα1 + G2-S16 treatments were observed compared to Tα1 (Figure 3(C.5)). No further changes were observed in pDCs for any studied marker. 

Thus, it seems that the combined administration of both treatments enhances the activation and maturation of DCs during HCMV infection.

### 2.4. Treatment with Tα1 and PCDs Modulates the Expression of Co-Receptors in CD4+ and CD8+ T Cells, as Well as the Production of Pro-Inflammatory Cytokines

**Figure 4:**
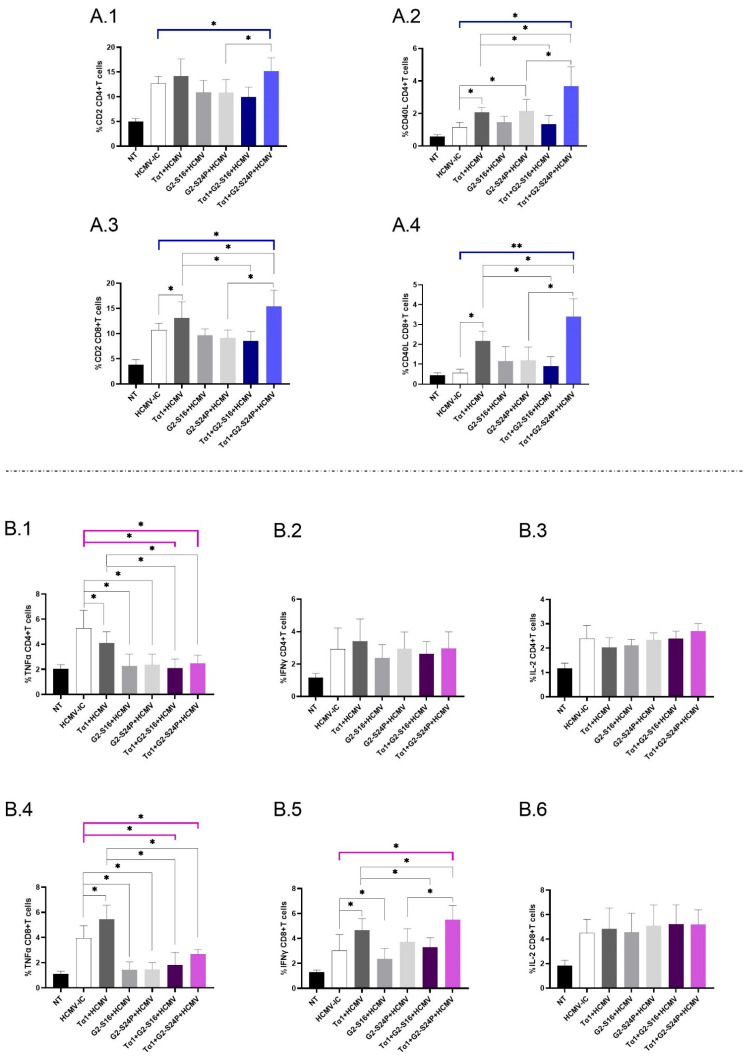
Immunophenotyping and cytokine production in CD4+ and CD8+ T cells in Tα1 and PCDs co-treatment. PBMCs (300,000 cells) were pre-treated with Tα1, PCDs and their combinations, infected with HCMV 2 h later, and then cultured for 48 h in culture medium at 37 °C/5% CO_2_. The expression of co-receptor marker and intracellular cytokines production in T cells were analyzed by multiparametric flow cytometry. Bar graphs represent the expression of co-receptors CD2 and CD40-L in CD4+ T cells (**A.1**,**A.2**) and in CD8+ T cells (**A.3**,**A.4**); percentage of TNFα, IFNγ and IL-2 cytokine production in CD4+ T cells (**B.1**–**B.3**) and in CD8+ T cells (**B.4**–**B.6**). NT: Non-treated control; HCMV-IC: HCMV Infection control; Tα1 + HCMV: Tα1 treated and HCMV infected condition; G2-S16 + HCMV: G2-S16 treated and HCMV infected condition; G2-S24P + HCMV: G2-S24P treated and HCMV infected condition; Tα1 + G2-S16 + HCMV: Tα1 + G2-S16 treated and HCMV infected condition; Tα1 + G2-S24P + HCMV: Tα1 + G2-S24P treated and HCMV infected condition. The mean values (mean ± SD) of at least three independent experiments are shown (* *p* ≤ 0.05; ** *p* ≤ 0.01). Abbreviations: PBMCs: Peripheral Blood Mononuclear Cells; PCDs: polyanionic carbosilane dendrimers; HCMV: Human Cytomegalovirus; Tα1: Thymosin-alpha-1.

To investigate the immunomodulatory effects of treatment with Tα1 + PCDs in CD4+ and CD8+ T cells during HCMV infection, we evaluated the expression of CD2 and CD40L, as well as the production of TNFα, IFNγ and IL-2 using multiparametric cytometry. When analyzing the immunophenotyping results for CD4+ T cells, a significant increase in CD2 expression was observed in Tα1 + G2-S24P compared to HCMV-IC, and Tα1 + G2-S24P showed higher expression than G2-S24P alone (Figure 4(A.1)). Similar results were observed for CD40L expression but, additionally, both Tα1 + PCDs behaved inversely; Tα1 + G2-S24P showed higher expression compared to Tα1, while Tα1 + G2-S16 showed lower expression. Additionally, increased CD40L expression was observed between G2-S24P and Tα1 alone compared to HCMV-IC (Figure 4(A.2)).

Regarding CD8+ T cells, a significant increase in CD2 expression was observed for Tα1+ G2-S24P compared to HCMV-IC. In addition, increased expression was noted in both Tα1 + PCDs treatments compared to Tα1 alone, as well as between Tα1 + G2-S24P treatment and G2-S24P alone. Moreover, Tα1 exhibited increased CD2 expression compared to HCMV-IC (Figure 4(A.3)). Similar results were observed in CD40L expression (Figure 4(A.4)). No further changes were observed in any treatment condition.

Analyzing cytokine production in CD4+ T cells revealed a significant decrease in TNFα production for both Tα1 + PCDs treatments and each Tα1, G2-S16 or G2-S24P alone compared to HCMV-IC. Lower TNFα production was also observed between both Tα1 + PCDs treatments and Tα1 alone (Figure 4(B.1)). Nonetheless, no further changes were observed for IFNγ or IL-2 production for any treatment conditions (Figure 4(B.2,B.3)). 

Concerning cytokine production in CD8+ T cells, similar results to those reported for CD4+ T cells in TNFα production were noted except for a significant increase in Tα1 treatment compared to HCMV-IC (Figure 4(B.4)). Additionally, a significant increase in IFNγ production was observed for Tα1 + G2-S24P treatment compared to HCMV-IC. Moreover, higher IFNγ production in Tα1 + G2-S24P treatment and lower IFNγ production in Tα1 + G2-S16 was observed compared to Tα1 alone. In addition, increased IFNγ level was noted in Tα1 + G2-S24P compared to G2-S24P. Furthermore, the results indicated an increase in Tα1 and a decrease in G2-S16 treatments compared to HCMV-IC (Figure 4(B.5)). Finally, no significant changes were observed for any treatments in IL-2 production (Figure 4(B.6)). 

Therefore, the combination of Tα1 and PCDs, particularly the Tα1 + G2-S24P treatment, could enhance the interaction between DCs and T cells by increasing co-receptor expression and promoting IFNγ production.

### 2.5. Treatments with Tα1 and PCDs Increase Treg Activation during HCMV Infection

To evaluate the effect of Tα1 and PCDs combined treatment on Treg cells during HCMV infection, Treg frequency (CD3 + CD45RA-CD4 + CD25 + CD127-FoxP3+), CD57 and CD31 were analyzed using multiparametric cytometry. 

**Figure 5:**
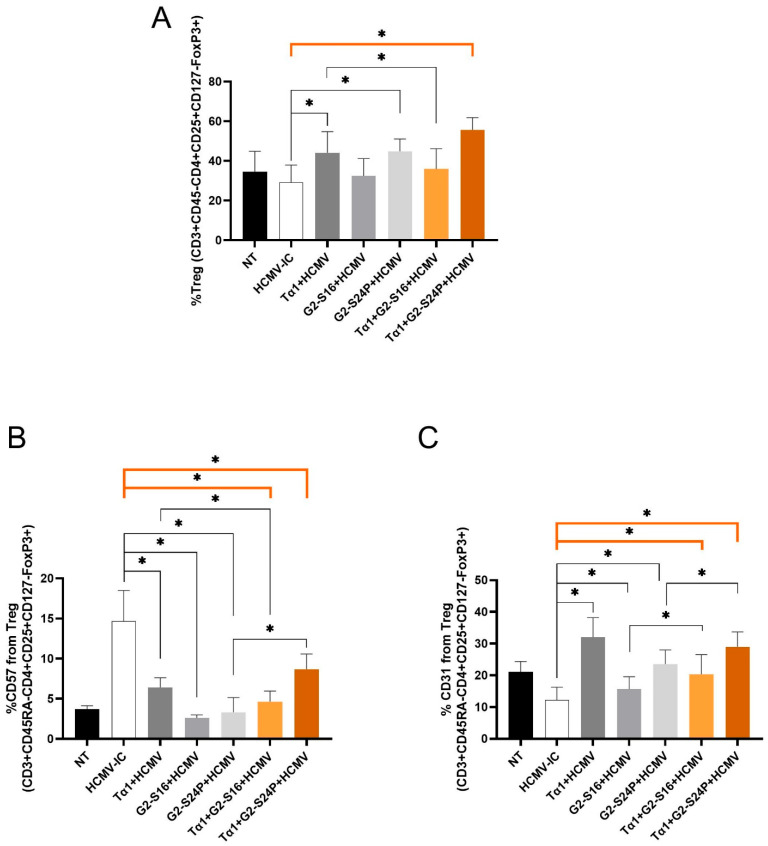
Immunophenotyping of Treg during HCMV infection in Tα1 and PCDs co-treatment. PBMCs (300,000 cells) were pre-treated with Tα1, PCDs and their combinations, infected with HCMV 2 h later and then cultured for 48 h in culture medium at 37 °C/5% CO2. Bar graphs represent percentage of Treg frequency (CD3 + CD45RA-CD4 + CD25 + CD127-FoxP3+) (**A**) and expression of senescence CD57 (**B**) and recent thymic emigrants CD31 (**C**) markers in Treg analyzed by multiparametric flow cytometry. NT: Non-treated control; HCMV-IC: HCMV Infection control; Tα1 + HCMV: Tα1 treated and HCMV infected condition; G2-S16 + HCMV: G2-S16 treated and HCMV infected condition; G2-S24P + HCMV: G2-S24P treated and HCMV infected condition; Tα1 + G2-S16 + HCMV: Tα1 + G2-S16 treated and HCMV infected condition; Tα1 + G2-S24P + HCMV: Tα1 + G2-S24P treated and HCMV infected condition. The mean values (mean ± SD) of at least three independent experiments are shown (* p ≤ 0.05). Abbreviations: PBMCs: Peripheral Blood Mononuclear Cells; PCDs: polyanionic carbosilane dendrimers; HCMV: Human Cytomegalovirus; Tα1: Thymosin-alpha-1; Treg: regulatory T cells.

The results demonstrated a significant increase in Treg frequency with Tα1 + G2-S24P treatment, Tα1, and G2-S24P alone compared to HCMV-IC, while a decrease was observed with Tα1 + G2-S16 treatment compared to Tα1. Furthermore, both Tα1 and G2-S24P treatments exhibited higher Treg frequency than HCMV-IC (Figure 5A). Conversely, a significant decreased CD57 expression was evident with both Tα1+ PCDs treatments and with each individual Tα1, G2-S16 and G2-S24P treatment compared to HCMV-IC. Additionally, reduced CD57 expression was observed in Tα1 + G2-S16 treatment compared to Tα1 alone; however, higher CD57 expression was observed in Tα1 + G2-S24P treatment compared to G2-S24P alone (Figure 5B). Furthermore, significantly increased CD31 expression was noted with both Tα1+ PCDs treatments and with each individual Tα1, G2-S16 and G2-S24P treatment compared to HCMV-IC. Finally, higher expression CD31 levels were also observed in Tα1 + PCDs treatments compared to each PCD treatment alone (Figure 5C). These findings suggest that the combination of Tα1 with PCDs enhances Treg functionality while reducing senescence. 

## 3. Discussion

The limitations of traditional antiviral therapies against HCMV infection have led to the development of novel treatments, including genetic, cellular and immune therapies [20]. Among these alternatives, dendrimers have demonstrated efficacy against different pathogens, including HCMV [21,22,23]. Our study highlights the combined immune effect of PCDs G2-S16 and G2-S24P with the immunomodulatory capacity of Tα1, showing their ability to enhance the functionality of DCs as antigen-presenting cells (APCs) and subsequently boost the activation of T cells, including Treg, promoting an adaptive response against HCMV infection. Moreover, this suggests a potential synergistic effect between Tα1 and PCDs, implying that the enhanced immune response results in increased effectiveness in the fight against the virus, even more than for Tα1, G2-S16 or G2-S24P alone. 

The initial phase of our study aimed to confirm the biocompatibility of G2-S16 and G2-S24P in PBMCs, in line with findings from prior studies [24]. As expected, cytotoxic assay, performed by MTT, revealed a dose-dependent reduction in mitochondrial activity caused by G2-S16 and G2-S24P PCDs, but without explicit indication of cell death [25,26]. Nonetheless, LDH results indicated that concentrations of G2-S16 and G2-S24P PCDs exceeding 10µL induced dose-dependent cell membrane damage in PBMCs, corroborating the observations from the MTT assay. Furthermore, the presence of Tα1 does not appear to influence the biocompatibility of PCDs, as similar results are observed in both PCDs treatment alone and in combination with the hormone. PCDs are hyperbranched molecules capable of forming complexes via electrostatic interactions among negatively charged functional terminal groups [27]. The cytotoxicity of dendrimers is highly dependent on the amount and nature of these surface functional groups. Cationic dendrimers often exhibit greater toxicity, whereas their anionic and neutral homologues, exemplified by G2-S16 and G2-S24P, usually show minimal or no adverse effects [28].

The subsequent step was to determine the effect of Tα1 + PCDs combination on the immune response using flow cytometry. Successful control of HCMV infection or reactivation requires the establishment of specific adaptive immune responses in the patient [29]. Previous studies have illustrated that HCMV employs various strategies to evade the host immune system, such as blocking DC maturation, reducing expression of co-stimulatory molecules, and lowering MHC class I/II levels expression, subsequently diminishing T cell immune responses [30]. Our results indicated that the combination of Tα1 with PCDs upregulated CD40, CD80, CD58, and TIM-3, suggesting that co-treatment enhances DC activation and maturation. Moreover, we observed an increase in HLA-DR MFI, which could confirm that the activation and maturation of DCs induced by the co-treatments improve antigen presentation, thus preventing immune evasion by HCMV [31,32,33]. Notably, a more pronounced increase in these markers was observed with combined Tα1 + PCDs treatments compared to individual Tα1 or PCDs treatments, implying a synergistic effect between Tα1 and PCDs. As expected, these markers exhibited higher increments in pDCs, a subset involved in combating intracellular pathogens, such as viruses [34]. Additionally, it appears that Tα1 + G2-S16 treatment increases activation markers, while Tα1 + G2-S24P is associated with maturation markers. Despite the differences, Tα1 + PCDs treatments would increase HLA-DR, suggesting that both would act in enhancing the antigen presentation of DCs. Activated and mature DCs induced by Tα1+ PCDs treatments could counteract HCMVs’ ability to impede DCs maturation and inhibit MHC class II antigen presentation, resulting in a more effective immune response.

Interestingly, the Tα1 + G2-S24P treatment exhibited heightened expression of the co-receptors CD2 and CD40L in both CD4+ and CD8+ T cells. This elevated expression was notably higher in the Tα1 + G2-S24P treatment compared to G2-S24P or Tα1 administered individually, implying a synergistic effect. Conversely, G2-S16 treatment, either alone or in combination with Tα1, did not induce this effect. Moreover, these differences in T cell activation between both PCDs were observed in cell cultures by the presence of aggregates in Tα1 + G2-S24P treatment, a phenomenon not observed in Tα1 + G2-S16 treatment. In both Tα1 + PCDs treatments, there was a reduction in the number and the size of aggregates compared to the HCMV-IC, suggesting activation of PBMCs without reaching a state of cellular exhaustion [35,36].

Furthermore, examination of proinflammatory cytokine production indicated an IFNγ increase production in CD8+ T cells with the Tα1 + G2-S24P combination. HCMV employs evasion strategies, countering the host IFNγ response. Upon HCMV entry, the disruption of lipid rafts or the use of entry inhibitors blocks IFN induction, while leaving the production of several other cytokines unaffected [37]. Therefore, Tα1 + G2-S24P treatment might counteract this viral effect, enhancing T cell response against HCMV. Moreover, results indicated reduced levels of TNFα with Tα1 + PCDs treatment, as well as with G2-S16 and G2-S24P alone. Several studies have indicated the potential anti-inflammatory capabilities of dendrimers [38,39,40]. Consequently, PCDs treatment alone or combined with Tα1 could mitigate the production of inflammatory cytokines, thereby regulating the overexpression of these cytokines during HCMV infection.

Regarding Treg, our findings also revealed a synergistic effect of Tα1 when combined with the PCDs, especially in the combination of Tα1 +G2-S24P. Tα1 has been demonstrated to modulate IFN-I type responses in pDCs and activate an immune suppression pathway by promoting Treg differentiation. This mechanism could promote highly specific adaptive immune responses and prevent immunopathology resulting from over-stimulation [9]. Our results indicated that Tα1 + PCDs treatment elevated the amount and functionality of Treg by upregulating FoxP3 and CD31 and downregulating CD127. In addition, we observed diminished senescence by downregulating CD57. Reduced CD31 and increased CD127 expression have been associated with proliferation and activation of T cells following antigen priming, consequently reducing Treg-mediated suppression [41,42,43]. During the acute phase of infection, HCMV reduces CD31 expression, disrupting the Treg balance and promoting overstimulation and exhaustion of T cell responses [44]. However, HCMV also employs Treg generation as an immune evasion strategy, aiding in latency establishment and evading cytotoxic effects induced by CD8+ T cells [45]. Thus, the use of these treatments during the latency phase of infection might be subject to debate and more studies are needed [46]. Nevertheless, during the acute period, Tα1 + PCDs also mitigated Treg exhaustion, enhancing its functionality. Therefore, Tα1 + PCDs treatments seem to establish a balance between the cytotoxic effects of CD8+ T cells and Treg, potentially playing a beneficial role in acute HCMV infection or reinfection [47,48].

Despite the similar physicochemical properties shared between both dendrimers, there is a notable distinction between G2-S16 and G2-S24P PCDs. G2-S16 possesses a more flexible chemical structure and this characteristic potentially enables G2-S16 to interact more readily with viral or cellular proteins [18]. In fact, our results demonstrate that treatment with G2-S16 alone, or in combination with Tα1, presents an absence of morphological changes in PBMCs, correlating with a reduction in the fluorescence intensity of IE viral protein. It suggests that G2-S16 could inhibit virus replication and prevent the newly transcribed virus from infecting nearby cells, as observed in previous studies [49]. Moreover, the capacity of G2-S16 to impede viral replication in the early stages of infection could be associated with reduced co-receptor expression, intracellular cytokine production in T cells, and lower expression of functionality and senescence markers in Treg compared to G2-S24P observations. This suggests that the structural differences could entail different mechanisms of action. Therefore, G2-S16 exerts a more direct effect and, consequently, a proper innate response, via DCs, but a lower activation of the adaptive response, while G2-S24P would establish a stronger synergy with Tα1-mediated immunomodulation at the level of the innate and adaptive responses.

However, the specific immunological mechanism of action against HCMV for both dendrimers has not yet been elucidated. This implies that we remain uncertain whether the synergistic action observed in Tα1 + PCDs treatment arises from the effect exerted by PCDs, from the recognized immune effect of Tα1, or whether there are different mechanisms of action simultaneously. Nonetheless, further investigations are needed to fully understand the beneficial properties of this combination in the immunotherapy of HCMV infection.

In conclusion, our study highlights the potential benefits of combining Tα1 with PCDs in early HCMV infection and demonstrates their synergistic effects. This combination shows promise as an alternative therapeutic regimen for prophylaxis of HCMV infection in immunocompromised patients.

## 4. Materials and Methods

### 4.1. Cell Isolation 

Peripheral Blood Mononuclear Cells (PBMCs) from buffy coats were obtained from anonymous healthy blood donors donated by the Transfusion Centre of Madrid following national guidelines (*n* = 28) and collected in ethylenediaminetetraacetic acid (EDTA) using Ficoll (Ficoll-Paque TM PLUS) by density gradient centrifugation on blood collection day and used immediately. After isolation, PBMCs were resuspended in 10% RPMI medium (RPMI 1640 supplemented with 10% heat-inactivated fetal bovine serum (FBS), 100 U/mL penicillin G, 100 µL/mL streptomycin sulfate and 1% l-glutamine) (Biochrom AG, Berlin, Germany). Interleukin-2 (60 U/mL) (rhIL-2; Bachem AG, Bubendorf, Switzerland) was added to PBMCs.

### 4.2. Reagents and Virus

#### 4.2.1. Polyanionic Carbosilane Dendrimers (PCDs)

G2-S16, with a silicon core and 16 sulfonate groups in the periphery, and G2-S24P, with a polyphenolic core and 24 sulfonate groups in the periphery, were used. All dendrimers ranged between 1 and 20 nm, becoming larger as the generation of the dendrimers increased. PCDs were synthesized and analyzed according to methods reported by the Dendrimers for Biomedical Applications Group of University of Alcalá (Madrid, Spain) NMR spectroscopy data confirming the identity of the compounds, and their structure and synthesis are presented in our previous articles [16]. Stock solutions of dendrimers (2.5 and 5 mM) and subsequent dilutions to working concentrations were prepared in nuclease-free water (Promega, Madrid, Spain).

#### 4.2.2. Thymosin-Alpha-1 (Tα1)

Lyophilized Tα1 (MyBioSource, San Diego, CA, USA) was reconstituted in nuclease-free water (Promega, Madrid, Spain) with 0.1% BSA at stock concentration of 100 µg/mL. Subsequent dilution to working concentration was prepared at 50 ng/mL.

#### 4.2.3. Virus

The viral strain HCMVAD–169 (ATCC VR-538) was expanded and titrated in MRC-5 cell line by plaque assay with serial dilutions. Stock aliquots at 3.5 × 10^6^ PFU/mL were prepared by ultracentrifugation and stored at −80 °C.

### 4.3. Mitochondrial Activity Assay

The mitochondrial toxicity of G2-S16 and G2-S24P PCDs was tested by the 3-(4-5-dimethylthiazol-2-yl)-2,5-diphenyltetrazolium bromide (MTT) assay (Sigma, St. Louis, MO, USA) according to the manufacturer’s instructions. Briefly, 2.5 × 10^5^ cells/well of PBMCs were seeded in 96-well plates and pre-treated with the desired compounds for 48 h. at concentration range of 5–50 µM. After incubation, culture medium was discarded and 220 µL of a 1:11 MTT (5 mg/mL)/OptiMEM solution was added to cultured PBMCs. After 3 h, the supernatant was removed, and formazan crystals were dissolved in 200 µL DMSO (Sigma, St. Louis, MO, USA). The absorbance was read in a Berthold Plate Reader at 570 nm. All points were performed in triplicate. Absorbance values were interpreted as a measurement of cell viability. Concentrations resulted in cell viability above 80% [19]. DMSO 10% was used as a positive control of cell death. Non-treated PBMCs were used as a viability control.

### 4.4. Membrane Integrity Assay

Cellular toxicity was measured by the lactate dehydrogenase (LDH) assay CytoTox 96^®^ Non-Radioactive Cytotoxicity (Promega, Spain, Madrid) following the manufacturer’s instructions. Briefly, 2.5 × 10^5^ PBMCs were seeded in 96-well plates and treated with the desired compounds for 48 h at concentration range of 5–50 µM. After the incubation period, PBMCs were lysed in 0.9% Triton X-100 (Promega, Spain, Madrid) for 45 min at 37 °C and 50 µL of LDH reagent (Promega, Spain, Madrid) was added for 30 min at room temperature, protected from light. The absorbance was read in a Berthold Plate Reader at 490 nm. All points were performed in triplicate. Absorbance values were interpreted as a measurement of cell viability. Concentrations resulted in cell viability above 80% [19]. Non-treated PBMCs were used as a viability control.

### 4.5. Cell Stimulations and Immunofluorescence

After isolation, PBMCs were plated in 96-well round-bottom plates (3 × 10^5^ cells/well) and pre-treated with the PCDs at the concentration of 10 μM concentration and with Tα1 at a concentration of 50 ng/mL for 2 h at 37 °C and 5% CO_2_. Then PBMCs were infected with HCMV (300 PFU/well) and incubated for 48 h at 37 °C and 5% CO_2_. During PBMCs culture, we used 1 µg/mL of anti-CD28/CD49d, 0.7 µg/mL of monensin (BD Biosciences, Franklin Lakes, NJ, USA) and 10 µg/mL of brefeldin A (Biolegend, San Diego, CA, USA) at 37 °C/5% CO_2_. 

To further investigate the results obtained from the stimulated culture, confocal microscopy was performed on PBMCs. In brief, 3 × 10^5^ PBMCs were incubated in an 8-well removable chamber (ibidi GmbH, Gräfelfing, Germany) for 48 h under the conditions described previously. Following incubation, the medium was removed, and cells were fixed in 4% paraformaldehyde (PFA; Panreac, Barcelona, Spain) for 10 min, washed three times in PBS, and permeabilized with 0.1% Triton X-100 (Sigma-Aldrich, St. Louis, MO, USA) for 10 min. After incubation, PBMCs were washed three times in PBS, blocked with 1% bovine serum albumin (BSA, Sigma-Aldrich, St. Louis, MO, USA) and 0.1% Triton X-100 in PBS for 30 min, and incubated with HCMV immediate early primary antibody (IE) (Bio-Rad, Hercules, CA, USA) for 1 h. Following incubation, PBMCs were washed two times with PBS and incubated with ALEXA FLUOR^®^ 647 secondary antibody and phalloidin ALEXA FLUOR^®^ FITC-conjugated antibody for 1 h. Subsequently, PBMCs were washed three times in PBS, stained with DAPI (Sigma-Aldrich, St. Louis, MO, USA) for 10 min. Finally, the silicone mold was removed, and the sample was mounted with a glass cover and analyzed using a Leica TSC SPE confocal microscope (Leica Microsystems, Wetzlar, Germany). Fluorescence was analyzed using LASX Office 1.4.5 27,713 (Leica Microsystems GmbH, Germany).

### 4.6. Flow Cytometry

For flow cytometry of dendritic cells (DCs), the immediately isolated PBMCs treated with PCDs (10μM) in combination with Tα1 (50 ng/mL) and subsequently infected with 300 PFU/mL of HCMV were washed with 3% PBS–BSA and stained with the following surface markers for 30 min: for viability, Aqua Blue Dead Cell Stain fixable LIVE/DEAD (Life Technologies, Waltham, MA, USA), markers for linage: anti-Lin2 FITC, anti-HLA-DR PerCP (clone L243) (BioLegend, San Diego, CA, USA) anti-CD11c AF700 (clone B-ly6) (BD Biosciences) anti-CD123 AF647 (clone #32703) (R and D Systems, Minneapolis, MN, USA); for activation: anti-CD40 Pe-Cy7 (clone 5C3), anti-CD80 BV421 (clone L307. 4), anti-CD58 BV605 (clone 1C3) (BD Biosciences); and for maturation anti-TIM3 PE (clone 7D3), (BD Biosciences). PBMCs were washed and fixed with 4% paraformaldehyde (PFA). Viable DCs were characterized by HLA-DR expression and myeloid dendritic cells (mDCs) and plasmacytoid dendritic cells (pDCs) were defined as HLA-DR + CD11c + CD123− and HLD-DR + CD11c-CD123+, respectively. Detailed information concerning the gating design can be seen in Supplementary Figure S1A. Isotype controls for CD40, CD80, TIM-3 and CD58 were included in each experiment.

For T cells’ immunophenotyping and intracellular cytokine staining, CD4+ and CD8+ T cells from the different treatment conditions were washed with 3% PBS-BSA and surface stained for 30 min using the following surface markers: for viability, LIVE/DEAD Fixable Aqua Blue Dead Cell Stain (Life Technologies), for linage anti-CD3 PerCP-Cy5.5 (clone SK7), anti-CD4 APC-Cy7 (clone RPA-54) (BD Biosciences), anti-CD8 PB (clone SK1) (Biolegend); for maturation anti-CD45RA ECD (clone 2H4) (Beckman Coulter); for recent thymic emigrants, anti-CD31 AF647 (clone WM59) (BD Biosciences); for senescence marker anti-CD57 FITC (clone TB01) (BD Biosciences); for receptor anti-CD25 BV421 (clone BC96) (Biolegend) and anti-CD127 PECy7 (clone A7R34) (Beckman Coulter); and for co-receptors anti-CD2 FITC (clone RPA-2.10) and anti-CD40L PE-Cy7 (clone 24–31) (BD Biosciences).

Additionally, one subset of PBMCs was washed and permeabilized, fixed with the Cytofix/Cytoperm kit (BD Biosciences), and stained intracellularly for 30 min with anti-TNF-α APC (clone Mab11), anti-IL-2 PE (MQ1-17H12), anti-IFN-γ BV605 (clone B27) and anti-CD3 PerCP-Cy5.5 (clone SK7) (BD Biosciences) for cytokine production analysis. In parallel, another subset of PBMCs was permeabilized with eBioScience FoxP3/Transcription Factor Staining Buffer Set (Thermo Fisher Scientific, Waltham, MA, USA) and stained with the intranuclear transcription marker FoxP3-PE (clone 259D/C7) (BD Biosciences) for Treg identification.

All of these were distributed into two different T-cell cytometry panels. T cells were defined as viable cells having low forward/side scatter and expressing CD3, and/or CD8/CD4. Detailed information concerning the gating design can be shown at Supplementary Figure S1B. Treg were defined by CD3 + CD4 + CD45RA-CD25 + CD127-FOXP3+ expression. Detailed information concerning the gating design can be shown at Supplementary Figure S1C. Isotype controls for CD2, CD40-L, CD31 and CD57 were included in each experiment.

Cells were analyzed with a CytoFLEX S cytometer (Beckman Coulter, Pasadena, CA, USA) and data were analyzed with FlowJo 8.7.7 (TreeStar, Ashland, OR, USA).

### 4.7. Statistical Analysis

Statistical analysis was performed using the Statistical Package for Social Science (SPSS 22.1; SPSS Inc., IBM, Endicott, NY, USA). The Wilcoxon test was used to analyze related conditions. Graphs were generated with GraphPad Prism version 9.0 (GraphPad Software, San Diego, CA, USA) (* *p* ≤ 0.05; ** *p* ≤ 0.01; *** *p* ≤ 0.001).

## Data Availability

All data generated or analyzed during this study are included in this published article.

## Supplementary Materials

The following supporting information can be downloaded at: https://www.mdpi.com/article/10.3390/ijms25041952/s1.
