# Supplementary figures and images for: Enhanced Immunomodulatory Effects of Thymosin-Alpha-1 in Combination with Polyanionic Carbosilane Dendrimers against HCMV Infection
Source: Int J Mol Sci. 2024 Feb 6;25(4):1952. doi: 10.3390/ijms25041952 (PMC10887890; doi:10.3390/ijms25041952)

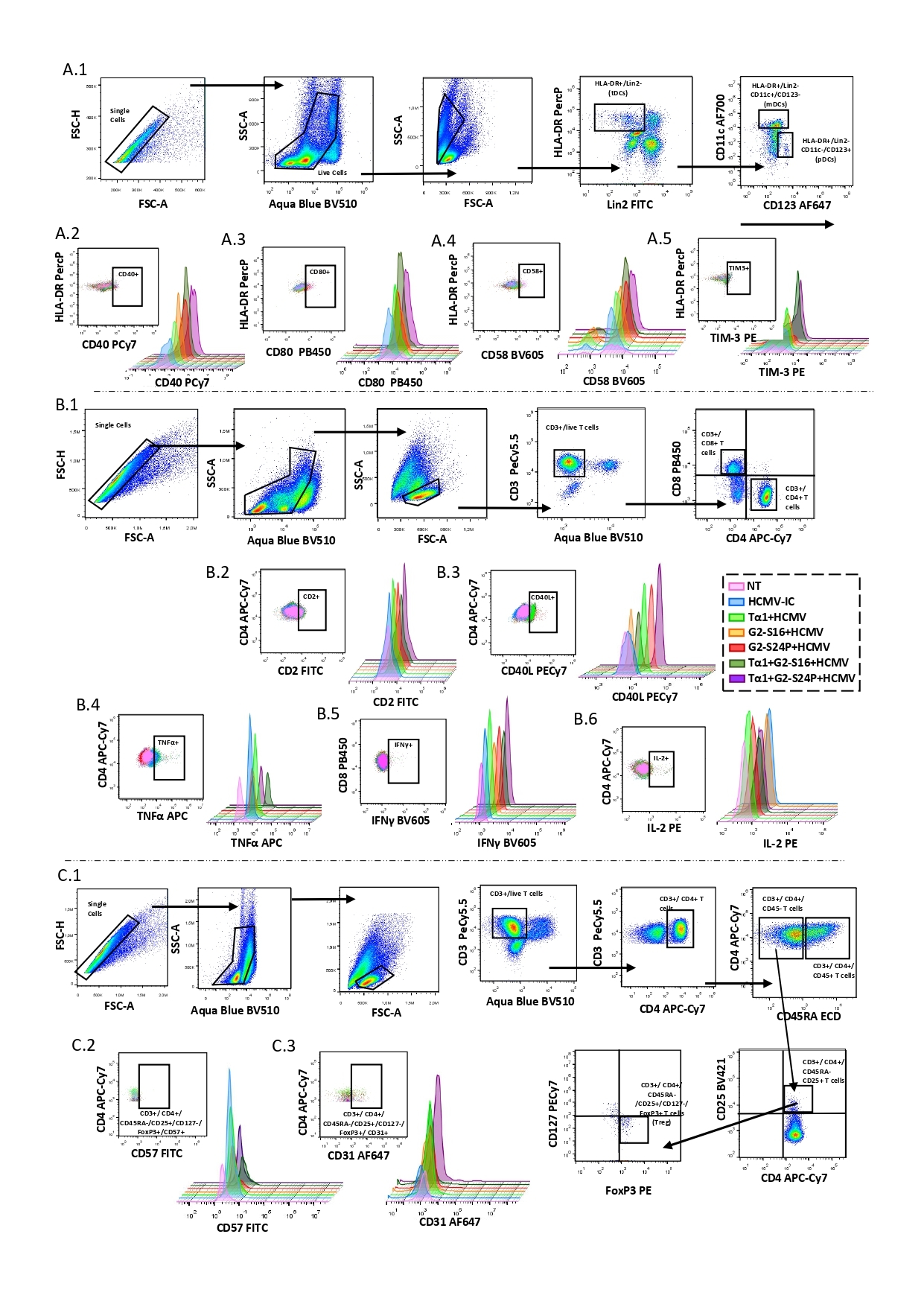

Supplement: Supplementary file 1 [file ijms-25-01952-s001.zip › Supplementary Figure 1.jpg]

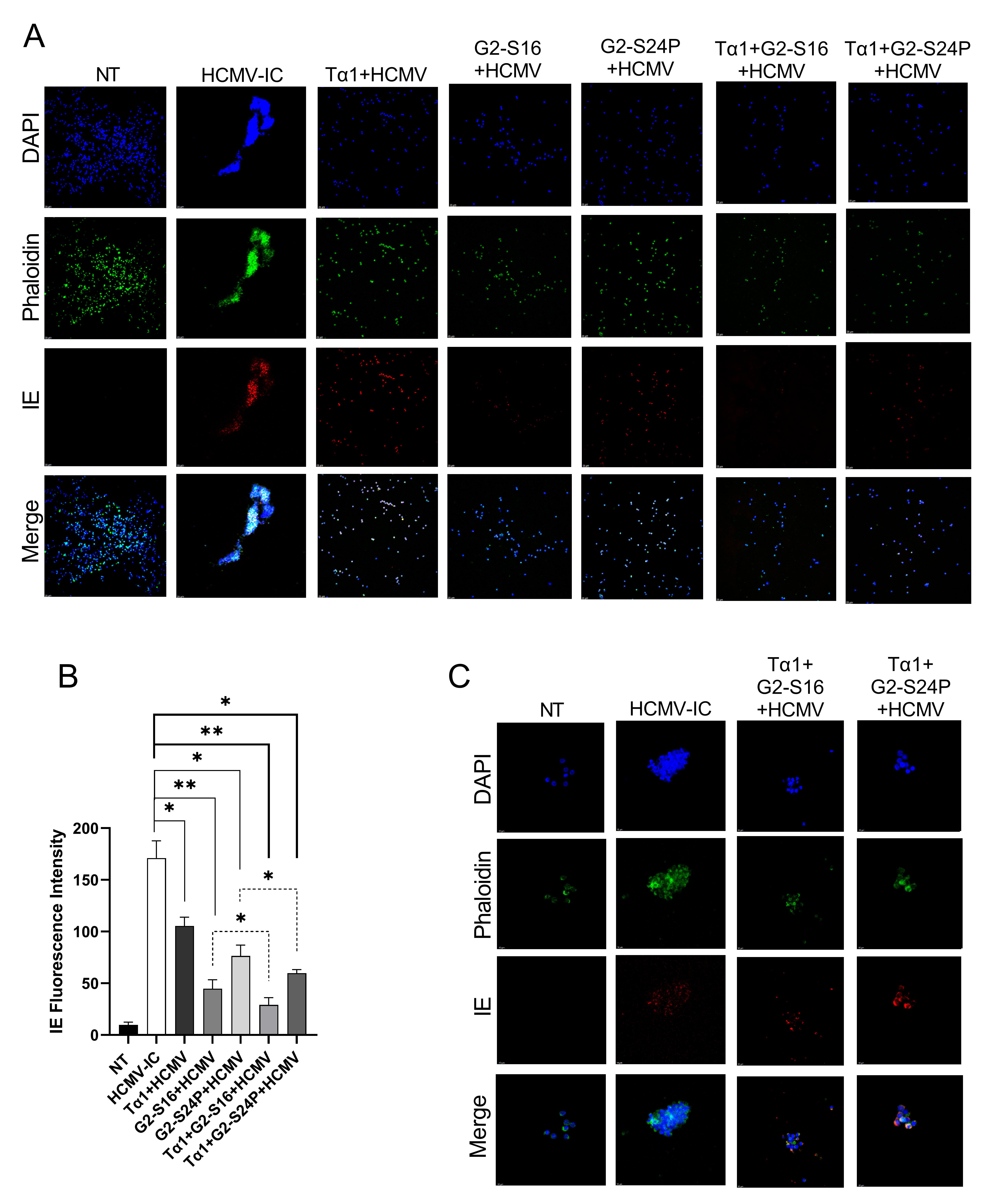

Supplement: Supplementary file 1 [file ijms-25-01952-s001.zip › Supplementary Figure 2.jpg]
